# An Integrated 5I Health Promotion Model for Enhancing Independence and Quality of Life Among Older Adults in Indonesia: A Community-Based Path Analysis Study

**DOI:** 10.3390/ijerph23030301

**Published:** 2026-02-28

**Authors:** Sri Suwarni, Agus Kristiyanto, Sapja Anantanyu, Anik Lestari

**Affiliations:** 1 Development Extension/Community Empowerment, Postgraduate School, Universitas Sebelas Maret, Surakarta 57126, Indonesia; sap_anan@staff.uns.ac.id; 2 Faculty of Sports, Universitas Sebelas Maret, Surakarta 57139, Indonesia; agus_k@staff.uns.ac.id; 3 Faculty of Medicine, Universitas Sebelas Maret, Surakarta 57126, Indonesia; aniklestari@staff.uns.ac.id

**Keywords:** health promotion model, independence, quality of life, older adults, path analysis, Indonesia

## Abstract

**Highlights:**

**Public health relevance—How does this work relate to a public health issue?**

- Population aging in low- and middle-income countries (LMICs) poses a major challenge due to limited health resources and the need for functional independence.
- There is a lack of integrated community-based health promotion models that address the complex interaction between personal beliefs and social support in urban settings.

**Public health significance—Why is this work of significance to public health?**

- The Integrated 5I Model provides an empirically validated framework (Identify, Inspire, Initiate, Integrate, and Impact) that achieves excellent statistical fit (CFI = 1.000).
- Participation in daily and social activities was found to be the strongest direct predictor of physical independence (β = 3.018, *p* < 0.001).
- Participation in social activities was found to have a significant direct impact on the quality of life among the elderly (β = 2.376, *p* = 0.003), highlighting the importance of psychosocial integration.

**Public health implications—What are the key implications or messages for practitioners, policy makers, and/or researchers in public health?**

- Health promotion for older adults in Indonesia is most effective when embedded within local spiritual-social structures and cultural social capital.
- Practitioners and policymakers should utilize the Pentahelix approach (government, academia, community, media, and private sector) to ensure the sustainability of active aging programs.

**Abstract:**

Population aging poses a growing public health challenge in low- and middle-income countries, including Indonesia. Functional independence is a key determinant of older adults’ quality of life, yet integrated community-based health promotion Models addressing this issue remain limited. This study developed and empirically validated an Integrated 5I Health Promotion Model (Identify, Inspire, Initiate, Integrate, and Impact) to enhance independence and quality of life among older adults in an urban Indonesian setting. A community-based cross-sectional survey was conducted among 240 older adults in Surakarta, Indonesia, using proportional cluster sampling from community activity groups. The integrated 5I Model was constructed based on the Health Belief Model, the Logic Model, and a pentahelix approach. The data were collected using a structured questionnaire comprising the Mini-Mental State Examination (MMSE), the Geriatric Depression Scale (GDS), the modified PASE (including religious activities), and an expanded WHOQOL-BREF (incorporating spirituality, freedom, and happiness). The data were analyzed using path analysis to examine direct and indirect relationships among internal and external factors, perceptions, participation, independence, and quality of life. The model demonstrated good structural fit and explained a substantial proportion of variance in independence and quality of life. Perception and participation played significant mediating roles between the internal and external factors and independence. Increased independence was significantly associated with improved quality of life among older adults. Participation showed the most substantial direct effect with physical independence (β = 3.018, *p* < 0.001), while independence was significantly associated with quality of life (β = 0.599, *p* < 0.001). Participation was also found to have a significant direct impact on the quality of life (β = 2.376, *p* = 0.003). The model demonstrated excellent fit (CFI = 1.000; RMSEA = 0.000; SRMR = 0.012). The Integrated 5I Health Promotion Model offers a pragmatic, scalable framework for community-based interventions to promote independence and quality of life among aging populations in urban low- and middle-income settings. This model has important implications for public health programs and policies targeting healthy and active aging.

## 1. Introduction

Population ageing is accelerating worldwide, particularly in low- and middle-income countries (LMICs), where demographic transitions occur alongside limited health and social care resources [1,2]. Maintaining physical independence has become a critical priority [3], as functional decline is strongly associated with increased healthcare utilization, long-term care dependency, and reduced quality of life [4,5]. Recent global reports continue to highlight that functional independence remains the most reliable predictor of successful ageing in diverse urban settings [6]. The World Health Organization emphasizes functional ability and healthy ageing as key outcomes for ageing populations, highlighting the need for effective community-based health promotion strategies [1].

Daily activities, such as household chores, leisure pursuits, employment-related activities, and social interactions or participation in religious events, all affect physical independence [7,8]. These activities contribute to mobility, strength, social interaction, and psychological well-being. Previous studies have demonstrated that regular participation in such activities is associated with better functional status and quality of life among older adults [8,9]. However, in many LMIC contexts, these activities remain underutilized within structured health promotion programs.

Despite growing evidence on the benefits of active ageing, existing health promotion interventions for older adults are often fragmented and narrowly focused on individual behaviors. Many programs emphasize physical activity alone without adequately addressing cognitive perceptions, social participation, or environmental and institutional support [8,9]. As a result, these interventions may have limited sustainability and impact, particularly in community settings where complex interactions between personal beliefs, social networks, and structural factors shape older adults’ behaviors.

Integrated and theory-informed frameworks are therefore needed to guide health promotion strategies for older adults. Behavioral theories such as the Health Belief Model offer insights into how perceptions relate to health-related behaviors [10,11]. The Logic Model supports the systematic planning, implementation, and evaluation of interventions [12,13]. In addition, multisectoral collaboration, as conceptualized by the pentahelix approach, is increasingly recognized as essential for addressing complex public health challenges, including healthy ageing, at the community level [14,15].

While previous health promotion frameworks have significantly contributed to elderly care, they have yet to fully incorporate cultural and religious dimensions, which are pivotal in shaping health-seeking behaviors in LMIC settings like Indonesia. This study addresses this gap by integrating these spiritual and community-based values into the 5I Model (Identify, Inspire, Initiate, Integrate, and Impact). In this study, perception refers to older adults’ subjective cognitive appraisal of their individual health status and the external social support available in their urban environment. Using structural path analysis, this study examines the relationships among internal and external factors, perception, participation, independence, and quality of life. The proposed model provides a practical and scalable framework for community-based health promotion and offers evidence relevant to healthy ageing initiatives in LMIC settings. While recent studies have begun to explore community-based interventions in Indonesia [16], there remains a need for a model that integrates cultural-spiritual capital explicitly into a systematic pathway [17].

## 2. Materials and Methods

### 2.1. Study Design

This study employed a community-based cross-sectional analytical design to examine determinants of physical independence and quality of life among pre-older adults and older adults in Surakarta, Indonesia.

### 2.2. Study Setting and Population

Surakarta City, Central Java, Indonesia, is a metropolitan region with a well-established infrastructure for community-based senior health care. The research was carried out here. The target population consisted of community-dwelling adults aged 55 years and older, including pre-older adults (55–59 years) and older adults (60 years or older). The researchers excluded individuals with severe cognitive impairment or acute illness that prevented participation.

### 2.3. Sampling Technique and Sample Size

A proportional cluster sampling technique was applied. Community activity groups and elderly health posts (Posyandu Lansia) served as sampling clusters. Clusters were selected proportionally across administrative areas to ensure representation of different community settings. The researchers invited eligible participants within the selected clusters to participate.

A total of 240 respondents were selected using proportional cluster sampling. This sample size is considered adequate for path analysis [18], following the methodological recommendation of a 15:1 to 20:1 ratio of observations per estimated parameter [19]. With 6 main variables in the model, *n* = 240 provides sufficient statistical power for robust estimation.

### 2.4. Study Variables

The study examined relationships among internal factors, external factors, perception, participation, physical independence, and quality of life. Internal factors included individual characteristics such as age, sex, education, and health status. External factors reflected environmental and social support. Perception referred to beliefs and attitudes related to physical activity and ageing, such as knowledge, experience (including previous program exposure), perceived benefits, perceived barriers, and self-efficacy. Participation captured involvement in individual physical activities and in social and community engagement, including religious participation. Independence captured engagement in daily physical activities, including household, leisure-time, work-related, and religious activities. The researchers treated physical independence and quality of life as outcome variables.

### 2.5. Measurement Instruments

Data were collected using a structured questionnaire developed by adapting existing instruments and contextualizing them for older adults. The questionnaire comprised sections assessing internal and external factors, perception, participation, physical independence, and quality of life. Internal and external factors were measured using a 4-point Likert scale (1 = Strongly Disagree to 4 = Strongly Agree). The researchers assessed health participation using a combined format of Likert-type questions and binary (Yes/No) activity checklists. The study assessed all items using Likert-type scales and designed a structured yes-or-no questionnaire. Before data collection, language experts reviewed the instrument for clarity and cultural appropriateness. The study evaluated the reliability and validity of the constructs during data analysis.

The study assessed cognitive function using the Mini-Mental State Examination (MMSE), depressive symptoms using the Geriatric Depression Scale (GDS), physical activity independence using the Physical Activity Scale for the Elderly (PASE) expanded with items on frequency and role in religious activities, and quality of life using the WHOQOL-BREF, extended with three additional dimensions (freedom, spirituality, and happiness). All instruments have demonstrated acceptable validity and reliability in older adult populations, including Indonesian settings. Regarding the newly adapted spirituality dimension, we conducted validity testing through item-test correlations; the results confirmed that all items contributed significantly to the construct. Furthermore, the study verified the internal consistency of all instruments using Cronbach’s alpha (α), calculated with StataMP version 13, with interpretation criteria as follows [20]: 0.81–1.00 (very reliable), 0.61–0.80 (reliable), and 0.42–0.60 (quite reliable). The results indicated high reliability for the majority of variables: external factors (α = 0.8523), perception (α = 0.8911), and QoL (α = 0.8954). Although participation (α = 0.5069) and independence (α = 0.5400) exhibited lower internal consistency, the study retained these constructs given their critical theoretical significance in the 5I Model and positive feedback from face validity reviews. Most importantly, the overall reliability for the entire integrated instrument was very high (α = 0.9316), confirming its robustness for the final path analysis.

### 2.6. Data Collection Procedures

The researchers collected the data through face-to-face interviews conducted at participants’ homes or community meeting locations, depending on participants’ preferences, between July and August 2024. Each interview lasted approximately 30–60 min, and the researchers obtained written informed consent from all participants before data collection.

### 2.7. Data Analysis

The study used descriptive statistics to summarize participant characteristics and conducted path analysis to examine direct and indirect relationships among study variables in accordance with the proposed Integrated 5I Health Promotion Model. The study performed all statistical analyses using StataMP version 13, with statistical significance set at *p* < 0.05. Preliminary screening showed 100% data completeness among all 240 respondents; therefore, the study did not require any imputation for the missing data.

### 2.8. Ethical Considerations

The study was conducted in accordance with the Declaration of Helsinki and received ethical approval from the appropriate institutional review board. All participants provided informed consent, and the researchers strictly maintained data confidentiality.

## 3. Results

### 3.1. Participant Characteristics

A total of 240 respondents participated in this study. The participants were older adults aged 55–59 years and older adults aged 60 years or over. The majority were female, had completed basic to secondary education, and were living independently in the community. Most participants reported regular engagement in daily activities, including household tasks, leisure-time activities, religious activities, and, to a lesser extent, work-related activities. Table 1 presents detailed demographic characteristics.

### 3.2. Descriptive Statistics of Study Variables

Descriptive analysis showed moderate to high mean scores for perception and participation variables. Physical independence and quality-of-life scores indicated generally favorable functional status among participants. Table 2 presents summary statistics for internal factors, external factors, perception, participation, physical independence, and quality of life.

### 3.3. Bivariate Associations

Bivariate analysis demonstrated statistically significant associations between participation and physical independence, as well as between physical independence and quality of life (*p* < 0.05). Perception was also significantly associated with participation. Table 3 summarizes associations among the other variables.

### 3.4. Path Analysis Results

The researchers conducted a path analysis to examine the direct and indirect relationships among the study variables based on the Integrated 5I Health Promotion Model. As shown in Table 4, participation showed a significant direct effect on physical independence (β = 3.018, *p* < 0.05). Physical independence had a significant direct effect on quality of life (β = 2.376, *p* < 0.05).

**Figure 1:**
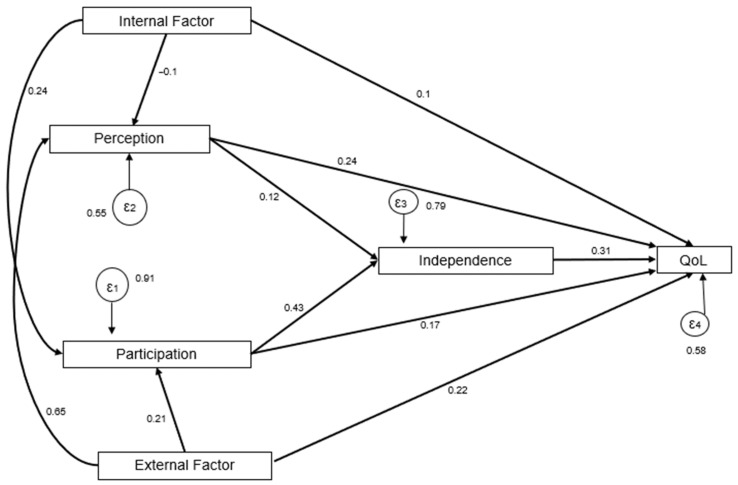
Path Analysis Diagram. The path analysis illustrates the structural relationships between predictor variables and health outcomes. Single-headed arrows represent direct paths, with standardized coefficients indicating the strength of the relationship.

Perception demonstrated an indirect effect on physical independence through participation. Internal and external factors were linked to physical independence indirectly via perception and participation pathways (Table 5). The total effects indicated that participation and physical independence were the strongest predictors of quality of life: Figure 1 and Table 6 present standardized path coefficients and significance levels.

The structural relationships between variables are detailed in Table 4. Based on these results, the model demonstrated an excellent fit, as shown in Table 7. The model achieved an exceptional structural fit, as evidenced by a χ^2^/df ratio of 0.334 (χ^2^ = 1.002, df = 3, *p* = 0.801), which is well within the recommended threshold of <3.0. This indicates that the hypothesized Model structure closely aligns with the empirical data. These indices, complemented by a Comparative Fit Index (CFI) of 1.000 and a Root Mean Square Error of Approximation (RMSEA) of 0.000, confirm that the hypothesized structural paths of the 5I Model precisely account for the observed variances in this urban sample. This statistical robustness suggests that the 5I framework offers a viable, empirically grounded approach for predicting health outcomes in similar community settings in Indonesia.

## 4. Discussion

**Figure 2:**
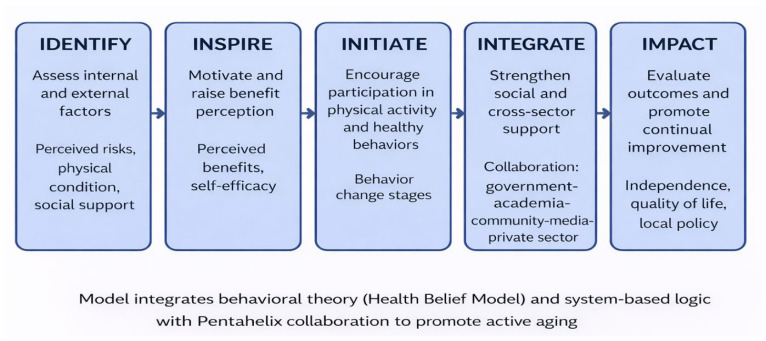
Integrated 5I Health Promotion Model for Active Aging. Identify: Assessment of internal and external factors. Inspire: Motivation stage focusing on benefit perception and self-efficacy. Initiate: Engagement in health-promoting behaviors and change stages. Integrate: Strengthening cross-sector support via the Pentahelix approach. Impact: Evaluation of independence, quality of life, and policy influence.

Our analysis empirically verified the Integrated 5I Health Promotion Model as a framework explaining how internal and external factors are associated with independence and quality of life among older adults engaged in health promotion activities at Posyandu Lansia. This study demonstrates a complex interaction between cognitive and behavioral components in fostering sustainable aging outcomes within the Indonesian context. The conceptual model of participants shown in Figure 2 is the Integrated 5I Health Promotion Model for Active Aging.

The model demonstrated exceptional statistical fit, highlighting its theoretical coherence and empirical strength in a critical public health context. While such values are rare in large-scale social research, in this context, they indicate that the 5I structural paths (Identify to Impact) fully capture the variance within the community-based activity groups in Surakarta. Consider that, for elderly populations already integrated into health posts (Posyandu), the progression from motivation (Inspire) to action (Initiate) and multisectoral support (Integrate) follows a highly predictable, linear trajectory.

Unique to the Indonesian context, our model shows that the “Initiate” and “Integrate” stages are heavily supported by religious and cultural social capital—activities such as communal prayers and pengajian serve as informal platforms for health promotion. Unlike Western models, which often focus on individual gym-based exercise [21,22], health promotion for Indonesian older adults must be “embedded” within these spiritual-social structures to ensure high sustainability and participation rates [23].

The “Integrate” component underscores the necessity of the Pentahelix approach. The study reveals that independence is not merely an individual outcome but a product of systemic support—ranging from government policy to local media and academic involvement. Without the “Integrate” stage, the transition from individual behavior change to long-term quality of life (Impact) becomes fragmented [14,24].

These findings are consistent with previous studies in LMIC contexts, which emphasize the importance of daily activity engagement and social participation in maintaining functional independence among older adults [21,22,23]. Compared to intervention-based studies in high-income countries, the Integrated 5I Model highlights a more context-sensitive and scalable approach suitable for resource-limited urban settings.

However, this study has limitations. The study drew the sample from active Posyandu participants, which may introduce a “healthy volunteer bias.” Therefore, the study has yet to test the effectiveness of the 5I Model among homebound or frail older adults. Future interventions should use the “Identify” stage to target those currently excluded from community groups specifically.

### 4.1. Principal Findings

This study developed and empirically tested the Integrated 5I Health Promotion Model to examine determinants of physical independence and quality of life among older adults in Indonesia. The findings demonstrate that participation in daily activities plays a central role in physical independence, which, in turn, is directly associated with quality of life. These results highlight the importance of engagement in everyday activities as a key pathway for promoting healthy ageing in community settings. The present study contributes to the growing body of evidence on healthy ageing by empirically demonstrating that participation in daily activities is a central pathway to physical independence, which, in turn, is associated with quality of life. This finding aligns with global ageing frameworks that emphasize functional ability rather than disease-centered outcomes as the core objective of ageing policies [1,3]. Recent evidence from Crocker et al. [6] further supports this policy focus on functional ability by emphasizing that maintaining physical independence is a cornerstone of sustainable aging in community-based settings.

### 4.2. Participation as a Driver of Independence and Quality of Life

Our findings demonstrate that participation is the single strongest predictor of physical independence (β = 3.018). In practice, this shows that older adults in urban Indonesia who remain socially active in community groups tend to maintain superior physical function. Our findings aligns with global evidence suggesting that daily social–physical engagement serves as a dual protective factor against both cognitive and functional decline [6,7].

The strong association between participation and physical independence is consistent with previous studies showing that engagement in daily and social activities reduces functional decline and disability among older adults [6,7,15]. Research from both high-income and low- and middle-income countries indicates that household activities, leisure participation, and social or religious engagement have meaningfully contributed to maintaining mobility and autonomy in later life [9,21,25].

Compared with structured exercise programs, daily activity-based participation is more sustainable and contextually appropriate for community-dwelling older adults, particularly in LMIC settings where access to formal exercise facilities may be limited [22,26]. Our research supports calls for broader conceptualizations of physical activity within public health interventions for ageing populations. Recent research by Agyemang et al. [27] similarly highlights that for older adults in LMIC regions, the collaboration between social networking and physical activity is essential for preventing functional decline. Furthermore, our study reveals that participation not only drives physical autonomy but also acts as a primary determinant of overall well-being. Specifically, participation in social activities was found to have a significant direct impact on the quality of life (β = 2.376, *p* = 0.003). This suggests that for the elderly in urban Indonesia, being socially integrated through community groups provides a psychological and social boost that directly translates into a higher perceived quality of life.

### 4.3. Cultural Integration and Spiritual Capital (Novelty)

A unique dimension identified in this validation of the 5I Model is the crucial role of the “Initiate” and “Integrate” stages when rooted in local values. In Surakarta, religious structures such as pengajian (communal religious gatherings) inextricably link health-promoting behaviors to community life. Unlike Western health promotion Models that often emphasize individualistic gym-based interventions [21], the 5I Model in Indonesia leverages “spiritual capital” as a catalyst. This cultural embedding enhances self-efficacy and the long-term sustainability of health behaviors among the elderly [9,23]. The integration of local wisdom into health frameworks, as suggested by Muhammad. [28], reinforces our finding that spiritual gatherings serve as a primary vehicle for health initiation in the Indonesian context.

### 4.4. The Pentahelix Approach for Sustained Impact

The “Integrate” component underscores the necessity of a Pentahelix approach. The study reveals that independence is not merely an individual achievement but a product of systemic collaboration encompassing academic mentorship, government policy, and local media advocacy. Without this multisectoral integration, health interventions remain fragmented and fail to achieve a permanent improvement in the quality of life (Impact) [14,23].

### 4.5. The Role of Perception in Shaping Participation

The indirect effect of perception on physical independence through participation underscores the importance of cognitive and motivational factors in health behavior. This finding aligns with behavioral theories suggesting that individuals’ beliefs about ageing, physical activity, and perceived benefits influence their willingness to engage in active behaviors [11]. Previous studies have similarly reported that positive perceptions of ageing and physical activity are associated with higher levels of participation and functional outcomes among older adults [29,30]. By empirically positioning perception as an intermediate pathway, the Integrated 5I Model extends prior work that often examines beliefs and behaviors separately, offering a more coherent explanation of how cognitive factors translate into functional outcomes.

### 4.6. Independence as a Determinant of Quality of Life

Physical independence emerged as a key determinant of quality of life, consistent with extensive literature demonstrating that functional ability strongly predicts well-being, social participation, and perceived life satisfaction among older adults [4,9,24]. Studies across diverse cultural contexts have shown that loss of independence is associated with poorer mental health outcomes and increased healthcare utilization [31,32]. Our findings align with international comparative data; for instance, Hui et al. [33] and Yılmaz et al. [34] demonstrated that, across diverse urban environments, social participation consistently acts as a buffer against the negative impacts of physical aging on life satisfaction.

These findings reinforce international policy recommendations that prioritize functional independence as a primary outcome of healthy ageing strategies [1,3].

### 4.7. Implications of the Integrated 5I Health Promotion Model

The Integrated 5I Health Promotion Model advances existing frameworks by integrating behavioral, social, and contextual determinants within a single empirically tested pathway. While previous studies have examined individual components such as physical activity or social participation in isolation [18,25], this study provides evidence of their interconnected effects on independence and quality of life.

Notably, the Model’s emphasis on community-based participation and multisectoral support aligns with contemporary public health approaches that advocate for scalable and sustainable interventions to address population ageing, particularly in resource-constrained settings [14,22,23]. Consider is consistent with the work, scalable Models in resource-limited areas must leverage existing community networks to ensure long-term program efficacy [35].

### 4.8. Strengths, Limitations, and Future Directions

Despite the model’s robust fit, this study has several limitations—first, the focus on active Posyandu Lansia participants in an urban setting. Consider may introduce a “healthy volunteer bias”. Future research should apply the 5I framework to homebound or socially isolated older adults to test the model’s consistency across diverse socio-environmental variables. Second, the sample size of 240, while statistically acceptable for the current path Model, represents a lower limit; thus, future studies should involve larger cohorts to increase the stability.

Additionally, several biases may affect the findings: (1) social desirability bias due to face-to-face interviews; (2) recall bias regarding physical activity levels (PASE); (3) selection bias. To minimize this bias, the study conducted home visits for participants who were unable to attend the community centers. Statistical analysis (Table 2) showed that home-visited participants had significantly lower independence scores (16.48 ± 4.08) than those at community centers (20.37 ± 5.71; *p* = 0.001), confirming that the study captured a broader spectrum of functional status among the elderly. Nevertheless, the study recruited the majority of the sample (n = 217) from active urban health centers (Posyandu), which may slightly overrepresent more socially active older adults; moreover, the study may be subject to standard method variance due to the use of self-reported questionnaires at a single point in time.

### 4.9. Public Health Implications

From a public health perspective, the findings suggest that promoting engagement in daily activities may represent a cost-effective strategy to enhance independence and quality of life among older adults. Health promotion programs that incorporate perception-building, participation facilitation, and community support may be particularly effective in LMIC contexts. The Integrated 5I Model provides a structured yet flexible framework that can inform community-based healthy ageing initiatives.

## 5. Conclusions

This study demonstrates that participation in daily activities is a key determinant of physical independence among older adults, which in turn is directly associated with quality of life. The path analysis results indicate that participation and physical independence function as central pathways linking individual and contextual factors to well-being in later life. These findings support the importance of community-based engagement as a feasible strategy for promoting healthy ageing.

This model provides an empirically supported framework that integrates perception, participation, and social support into a coherent pathway for enhancing independence and quality of life. By emphasizing everyday activities rather than structured exercise alone, the model offers practical relevance for implementation in resource-limited community settings. These findings suggest that the 5I Model can be strategically implemented by public health policy makers and community health practitioners, such as those involved in Integrated Healthcare Posts (Posyandu), to create culturally sensitive and spiritually aligned health promotion programs for urban elderly populations. The model’s applicability is particularly relevant in urban, low-income communities with active community-based health networks similar to the study environment. Future studies should use longitudinal designs and diverse populations to further validate and refine the 5I Model and to examine its temporal effects.

The Integrated 5I Model (Identify, Inspire, Initiate, Integrate, Impact) provides a pragmatic and culturally sensitive framework for health promotion in Indonesia. Participation remains the key determinant of independence and directly correlates with the quality of life in old age.

## Figures and Tables

**Table 1:** Sociodemographic and Psychological Characteristics of Participants (N = 240).

Variable/Category	Sub-Category	%	*n*
Age (years)	Mean ± SD: 66.4 ± 7.2	—	240
Gender	Female	62.5%	150
Marital Status	Married	58.8%	141
	Widowed/Divorced	38.7%	99
Occupational Status	Active	42.3%	102
	Retired	57.7%	138
Cognitive function (MMSE)	≥24 (Normal)	63.0%	151
Depression (GDS ≤ 10)	Low–Moderate	69.1%	166

**Table 2:** Respondent Characteristics by Interview Location (n = 240).

Characteristics	Total (n = 240)	Community Centers (n = 217)	Home Visits (n = 23)	*p*-Value
Age (Mean ± SD)	68.16 ± 6.89	68.07 ± 7.05	69.00 ± 5.22	0.446
Gender, n (%)				0.869
- Female	191 (79.6%)	173 (79.7%)	18 (78.3%)	
- Male	49 (20.4%)	44 (20.3%)	5 (21.7%)	
Health Status scores				
- Independence (Mean ± SD)	19.96 ± 5.69	20.37 ± 5.71	16.48 ± 4.08	0.001
- Quality of Life (Mean ± SD)	174.85 ± 14.21	175.12 ± 13.74	172.04 ± 20.73	0.951

**Table 3:** The Spearman Correlation Matrix Among the Six Major Constructs (N = 240).

Variable	*r* *p*	Internal Factors	External Factors	Perception	Participation	Independence
External Factors	*r*	−0.0591				
	*p*	0.3623				
Perception	*r*	−0.1756	0.5502			
	*p*	0.0064	0.0000			
Participation	*r*	0.2464	0.2106	0.1397		
	*p*	0.0001	0.0010	0.0305		
Independence	*r*	0.0288	0.1671	0.2193	0.4223	
	*p*	0.6573	0.0095	0.0006	0.0000	
QoL	*r*	0.0906	0.4486	0.3683	0.3649	0.4232
	*p*	0.1620	0.0000	0.0000	0.0000	0.0000

**Table 4:** Structural Direct Effects.

Endogenous Variable		Exogenous Variable	Path Coefficient (β)	Error Std.	z-Value	*p*-Value	CI 95%	
							Lower	Upper
Participation	←	Internal Factors	0.130	0.033	3.91	0.000	0.065	0.195
	←	External Factors	0.038	0.011	3.37	0.001	0.016	0.060
Independence	←	Participation	3.018	0.410	7.36	0.000	2.214	3.822
	←	Perception	0.120	0.060	2.00	0.045	0.003	0.237
QoL	←	Participation	2.376	0.790	3.01	0.003	0.827	3.926
	←	Perception	0.495	0.135	3.67	0.000	0.231	0.760
	←	Independence	0.599	0.108	5.52	0.000	0.386	0.811
	←	Internal Factors	0.757	0.384	1.97	0.049	0.004	1.510
	←	External Factors	0.543	0.167	3.24	0.001	0.215	0.671

Note: The arrows (←) indicate the direction of the relationship in the path analysis, where the variable on the right (exogenous/predictor) influences the variable on the left (endogenous/outcome).

**Table 5:** Structural Indirect Effects.

Endogenous Variable		Exogenous Variable	Path Coefficient (β)	Error Std.	z-Value	*p*-Value	CI 95%	
							Lower	Upper
Independence	←	Internal Factors	0.348	0.119	2.93	0.003	0.115	0.581
	←	External Factors	0.212	0.060	3.52	0.000	0.094	0.330
QoL	←	Participation	1.807	0.246	7.36	0.000	1.325	2.288
	←	Perception	0.072	0.036	2.00	0.045	0.002	0.142
	←	Internal Factors	0.334	0.206	1.63	0.104	−0.069	0.737
	←	External Factors	0.621	0.129	4.80	0.000	0.368	0.874

Note: The arrows (←) indicate the direction of the relationship in the path analysis, where the variable on the right (exogenous/predictor) influences the variable on the left (endogenous/outcome).

**Table 6:** Structural Total Effects.

Endogenous Variable		Exogenous Variable		Path Coefficient (β)	Error Std	z-Value	*p*-Value	CI 95%	
								Lower	Upper
Participation	←	Internal Factors	0.130		0.033	3.91	0.000	0.065	0.195
	←	External Factors	0.038		0.011	3.37	0.001	0.016	0.600
Independence	←	Participation	3.018		0.410	7.36	0.000	2.214	3.822
	←	Perception	0.120		0.060	2.00	0.045	0.003	0.237
	←	Internal Factors	0.348		0.119	0.93	0.003	0.115	0.581
	←	External Factors	0.212		0.060	3.52	0.000	0.094	0.330
QoL	←	Participation	4.183		0.828	5.05	0.000	2.560	5.805
	←	Perception	0.567		0.140	4.06	0.000	0.293	0.841
	←	Independence	0.599		0.108	5.52	0.000	0.386	0.811
	←	Internal Factors	1.091		0.408	2.67	0.008	0.291	1.882
	←	External Factors	1.164		0.141	8.26	0.000	0.888	1.440

Note: The arrows (←) indicate the direction of the relationship in the path analysis, where the variable on the right (exogenous/predictor) influences the variable on the left (endoge-nous/outcome).

**Table 7:** Goodness-of-Fit (GOF) Indices.

Fit Index	Value	Interpretation
Chi-square (χ^2^) ms(3)	1.002	Not significant (*p* = 0.801), Model is consistent with the data
χ^2^/df	0.334	Excellent fit (Threshold < 3.0)
RMSEA (≤0.05)	0.000	(90% CI = [0.000–0.069]), Excellent fit
*p* (RMSEA ≤ 0.05)	0.904	High probability of a good fit
CFI (≥0.95)	1.000	Perfect comparative fit
TLI	1.027	>1, Superior to baseline Model
SRMR (≤0.08)	0.012	Excellent residual fit
AIC	8132.155	Moderate Model complexity
BIC	8198.287	Parsimonious, predictive Model structure

## Data Availability

The data presented in this study are available on reasonable request from the corresponding author. The researchers did not publicly archive the data due to privacy concerns and ethical restrictions on the use of participants’ sensitive health information. However, the complete datasets and Stata code supporting the reported results of the path analysis are available from the corresponding author upon a reasonable request, justified request, subject to review by the institutional ethics committee.
